# Management of STEC Gastroenteritis: Is There a Role for Probiotics?

**DOI:** 10.3390/ijerph16091649

**Published:** 2019-05-12

**Authors:** Mario Giordano, Maria Elisabetta Baldassarre, Viviana Palmieri, Diletta D. Torres, Vincenza Carbone, Luisa Santangelo, Federico Gentile, Raffaella Panza, Federica Di Mauro, Manuela Capozza, Antonio Di Mauro, Nicola Laforgia

**Affiliations:** 1 Pediatric Nephrology and Dialysis Unit, Pediatric Hospital “Giovanni XXIII”, Bari 70123 Italy; mariogiordanobari@fastwebnet.it (M.G.); dilettatorres@libero.it (D.D.T.); enzacarbone2009@alice.it (V.C.); luisa.santangelo@hotmail.it (L.S.); fede_genti@msn.com (F.G.); 2 Neonatology and Neonatal Intensive Care Unit, Department of Biomedical Science and Human Oncology, “Aldo Moro” University of Bari, Bari 70124, Italy; mariaelisabetta.baldassarre@uniba.it (M.E.B.); viviana_palmieri@libero.it (V.P.); raffaella.panza@uniba.it (R.P.); manuelacapozza26@gmail.com (M.C.); nicola.laforgia@uniba.it (N.L.); 3 Department of Public Health, University of Naples Federico II, Napoli 80138, Italy; dimauro.federica@libero.it

**Keywords:** “Shiga-Toxigenic *Escherichia coli*” [Mesh], “Probiotics” [Mesh], “Microbiota” [Mesh], “Hemolytic-Uremic Syndrome” [Mesh]

## Abstract

Shiga toxin-producing *Escherichia Coli* (STEC) infections routinely run as a common gastroenteritis, but in many cases they may evolve towards hemolytic uremic syndrome (HUS). HUS is a rare disease characterized by microangiopathic hemolytic anemia, thrombocytopenia, and acute renal failure. Gut microorganisms have a fundamental impact on human physiology, because they modulate normal intestinal functions and play a pivotal role in influencing the local and systemic immune responses. Despite surveillance established in many countries and major progresses in the understanding of STEC-HUS mechanisms, no specific treatment is currently available. Targeting the gut microbiota could represent a new potential therapeutic strategy in STEC infection. In this paper, we reviewed the current knowledge about microbiota characteristics of patients with STEC infections, as well as in vitro and in vivo evidence of probiotic supplementation in managing STEC gastroenteritis and in HUS onset prevention.

## 1. Introduction

### 1.1. Shiga Toxin-Producing Escherichia Coli (STEC) Gastroenteritis and Hemolytic Uremic Syndrome

Hemolytic uremic syndrome (HUS) is a rare disease characterized by microangiopathic hemolytic anemia, thrombocytopenia, and acute renal failure. While “atypical HUS” (a-HUS) is due to defective complement system activation and regulation, “typical HUS” is associated with Shiga toxin-producing *Escherichia coli* (STEC) [1,2].

STEC contamination occurs after ingestion of contaminated food or water, person-to-person transmission, or contact with ruminants or a contaminated environment. 

After bowel colonization by STEC, diarrhea may occur quickly, which may be hemorrhagic in 85% of cases. Other symptoms may be abdominal pain, fever, and vomiting.

In 15% of cases, STEC infection evolves into HUS, which features a pathognomonic symptomatic triad: anemia, thrombocytopenia and acute kidney injury [3]. HUS is due to various strains of STEC [4], which may generate two major types of Shiga toxin (Stx): Stx1, a family consisting of Stx1, 1c, and 1d, and the more heterogeneous Stx2 family, comprising the variants Stx2c, 2c2, 2d, 2d-activatable, 2e, and 2f [5]. 

The type of Stx produced by *Escherichia coli* (*E. coli*) greatly influences the clinical outcome of the infection. While some Stx types are responsible for asymptomatic infections and uncomplicated diarrhea, Stx2, 2c, and 2d-activatable can cause hemorrhagic colitis and HUS by endothelial damage [6].

Endothelial cells are the primary target of the toxic effects of Stx, which trigger a cascade of signaling events resulting in loss of endothelial antiadhesive, anti-inflammatory, and thrombo-resistant properties [7].

Thrombotic microangiopathy is the final histological feature of HUS. It consists of thickening of arterioles and capillaries, swelling and detachment of endothelial cells from the basement membrane, and formation of fibrin- and platelet-rich thrombi that obstruct the microcirculation of different organs—including and predominantly the kidneys [8].

There is no effective prophylaxis and treatment available for STEC infections. Symptomatic and supportive therapy may prevent HUS onset [9,10], requiring the correction of anemia and the careful control of renal function, body fluids, and electrolyte disorders [11]. 

HUS occurs sporadically, in clusters and epidemics [12,13]. The incidence of STEC-HUS is about 2/100,000, with a peak of 6.1/100,000 in children <5 years of age [14,15]. Mortality rates are 1–4% in children during the acute phase of the disease [16].

Long-term sequelae (proteinuria, hypertension, chronic kidney disease, and neurological impairment) are estimated to occur in 25% of STEC-HUS patients four years later; 3% develop end-stage renal disease [17].

In Italy, between 2009 and 2014, the overall incidence was 0.59/100,000 in the pediatric age group (0–15 years), but in younger children (<5 years) incidence was three-fold higher (1.7/100,000). The mortality rate was 2.8%, while neurological involvement was described in 20.3%. Dialysis was required in 48% of affected children during the acute phase [18]. These data are substantially in line with those found in developed countries. Unfortunately, strong information about the same data in less developed countries, where childhood diarrheal diseases are endemic, is missing [19].

### 1.2. The Human Gut Microbiota

The gut microbiota is a complex ecosystem, consisting of approximately 5000 species of microorganisms [20]. It confers beneficial health effects to the host by adjusting the development of the gut [21], hindering the growth of pathogens [22], enhancing the immune system [23], impinging on energy homeostatic systems [24], and influencing the gut–brain axis [25].

Dysbiosis is characterized by the presence of an unbalanced gut microbial community with alterations in the composition and metabolic activities of the gut microbiota [26], and has been related to the pathogenesis of many diseases [27].

*E. coli* is a Gram-negative, facultative anaerobic, rod-shaped, coliform bacterium that is often found in the lower gut of warm-blooded organisms. It colonizes the gastrointestinal tract of animals and humans soon after birth. Its concentration in human feces has been estimated to be approximately 109 per gram, thus constituting about 1% of the total biomass in the large intestine [28,29,30].

Some *E. coli* strains have acquired specific virulence factors by means of mobile genetic elements, such plasmids, transposons, and bacteriophages. Thus, STEC contamination might be considered a dysbiosis [31]. 

A recent study, investigating changes in the intestinal microbiota composition of patients with STEC infection compared to healthy controls, found a lower abundance of bacteria of the *Bifidobacteriales* and *Clostridiales* orders in infected infants. These results represent the first evidence of changes occurring in the intestinal microbiota of children during STEC infection [32].

Increasing evidence suggests that gut microbiota manipulation could treat or even prevent some intestinal diseases [31,32,33,34]. 

The successes in treatment of *Clostridium difficile* diarrhea with microbiota manipulation recently increased the interest for this treatment in other diseases with proven disruption of gut microbiota, such as ulcerative colitis or metabolic syndrome [35]. 

In this paper, we reviewed the scientific evidence of gut microbiota manipulation with probiotic in STEC gastroenteritis. 

## 2. Methods

An exhaustive search for eligible studies was performed in PubMed, Embase, Medline, Cochrane library and Web of Science databases.

The following Medical Subject Headings (MeSH) were used: “Probiotics”[Mesh], “Shiga-Toxigenic Escherichia coli”[Mesh], “Microbiota”[Mesh], “Hemolytic-Uremic Syndrome”[Mesh].

Proper Boolean operators “AND” and “OR” were also included to be as comprehensive as possible. Additional studies were sought using references in articles retrieved from searches. 

Search limits were set for studies published between April 2009 and April 2019. As a result, a total of 37 papers were evaluated.

## 3. Probiotics

Probiotics are live microorganisms that, when administered in adequate amounts, confer health benefits to the host, and are mainly represented by lactic acid bacterial groups, such as *Bifidobacteria* and *Lactobacilli*, and yeasts, such as *Saccharomyces* [33].

In the last decades, probiotic supplementation for prevention and treatment of pediatric gastrointestinal diseases has been employed [34,35].

Probiotics are known to be effective in the management of acute gastroenteritis in children as an adjunct to rehydration therapy. 

In 2014, a position paper by the European Society for Pediatric Gastroenterology, Hepatology, and Nutrition (ESPGHAN) suggested *Lactobacillus GG* and *Saccharomyces boulardii* as effective strains for the management of acute gastroenteritis in children [36].

On the contrary, a recent multicenter, randomized, double-blind study comparing *Lactobacillus GG* with placebo [37] in children aged 3–48 months and diagnosed with acute intestinal infections, showed no differences between groups in prevention of moderate to severe diseases.

Currently, although probiotics are not routinely used in the management of STEC infections in humans, evidence from animal models infected with STEC suggests that they might represent an innovative preventive option [38].

In 2007, a systematic review suggested that the probiotic combination *L. acidophilus* NP51 and *P. freudenreichii* was effective in increasing animal resistance to STEC [39].

Furthermore, a 10-year review reported that probiotics could significantly reduce fecal STEC dismissal in ruminant animals, reservoirs of the pathogenic bacteria [40]. 

Beneficial effects of probiotics involve different mechanisms, such as competition with pathogens for adhesion to the epithelium and for nutrients, direct antagonism ascribed to production of specific molecules—such as bacteriocins—and, finally, immuno-modulation of the host [41]. 

In the last decade, different in vitro studies have assessed the antimicrobial potential of different probiotic strains against STEC.

In 2012, Mogna et al. demonstrated a significant in vitro inhibitory effect against the growth of STEC by five probiotic strains (*L. rhamnosus* LR04, *L. rhamnosus* LR06, *L. delbrueckii* subsp. *delbrueckii* LDD01, *L. pentosus* LPS01, and *B. breve* BR03) [42].

In 2013, Rund et al. tested the probiotic *Escherichia coli* strain Nissle 1917 (EcN) for antagonistic effects on three different STEC strain. Results showed that the probiotic EcN had very efficient antagonistic activity on the STEC strains tested [43]. In the same year, the probiotic strain *Enterococcus faecium* YF5, when cocultured with a STEC strain, demonstrated inhibition of the pathogen’s replication [44]. Additionally, the symbiotic use of *Lactobacillus fermentum* and *Bifidobacterium longum,* isolated from stools of healthy elderly individuals, demonstrated antimicrobial activity against STEC [45].

In 2014, Arena et al. demonstrated that *Lactobacillus plantarum* and *Lactobacillus fermentum* strains negatively influenced the adhesion of STEC [46].

In 2016, Dini et al. demonstrated the in vitro activity of a microbial mixture of five probiotic strains isolated from kefir grains (*L. plantarum*, *Lactococcus lactis*, *L. kefir*, *Kluiveromices marxianus* and *Saccharomyces cerevisiae*) in reducing the cytotoxic effect produced by in vitro STEC infection on epithelial Hep-2 cells [47]. Moreover, a study by Bian et al. demonstrated the antimicrobial potential of *Lactobacillus helveticus* KLDS against STEC [48].

However, it is important to note that the extent of probiotic protective capabilities seen in these experimental models is likely dependent on the probiotic strain used [49].

Several studies have also investigated the use of probiotics in animal models with an experimental STEC infection, and have shown the efficacy of various probiotic strains in preventing disease.

In 2010, Tsai et al. evaluated the immunomodulating activity of *Lactobacillus paracasei* subsp. *paracasei* NTU 101 in STEC-infected mice. They found a lower morbidity rate in mice supplemented with probiotic before and after STEC infection and speculated that this strain could be suitable for use as a probiotic in the prevention of STEC infection in humans [50].

Hostetter et al. investigated the efficacy of a Stx receptor mimic probiotic in a porcine model of HUS. In this study, piglets were inoculated with STEC and, after 24 hours, treated twice daily with a probiotic expressing an oligosaccharide receptor mimic for Stx2e in order to determine if the probiotic could reduce intestinal toxin levels. Results suggested that post-exposure treatment with a Stx-binding probiotic is effective in reducing intestinal Stx within the gut of a porcine model with a STEC infection [51].

The administration of probiotic agents to cattle has been proven to reduce their dismissal of STEC [52], thereby effectively diminishing the risk of transmitting these agents [53]. This effect might be due to the expression of molecules that mimic host cell receptors on the surface of harmless recombinant bacteria that can overcome digestive enzymes and other gastrointestinal environmental conditions [54].

To the best of our knowledge, only three studies have been conducted in the human-simulated digestive environment. In 2011, Etienne-Mesmin et al. demonstrated the antagonistic effects of the yeast strain *S. cerevisiae* CNCM I-3856 against STEC in the distal part of the small intestine [55]. A few years later, Thèvenot et al. demonstrated how the same yeast strain was responsible for a down regulation of Stx expression [56] and how the resident microbiota modulated pathogen infectivity in the human gastrointestinal tract [57].

Despite these promising findings, such studies have been conducted using oversimplified approaches and therefore results cannot be directly extrapolated to human situations.

A key question that clinical trial protocols in humans should address is whether probiotic supplementation might be advised either prophylactically to at-risk populations during STEC outbreaks or therapeutically to infected people. Even if evidence in this regard is still lacking, we suppose that early probiotic supplementation may interfere with the evolution of bloody diarrhea into HUS and could prevent systemic complications. The specific surveillance systems and protocols recently suggested by Freedman [58] may help clinicians in the early identification of children affected by STEC gastroenteritis, who might benefit from such preventive treatment. 

Finally, probiotic safety also needs to be investigated, as STEC infection leads to injury of epithelial gut barrier, which has been identified as a risk factor for adverse effects of probiotics [59]. Therefore, treating the acute phase of HUS with probiotics in critically ill patients may raise some concerns.

## 4. Conclusions

The leading cause of typical hemolytic uremic syndrome (HUS) in children is STEC infection, which can cause major outbreaks. Despite major progresses in the understanding of STEC-HUS mechanisms, no specific treatment is currently available. 

The results of the reviewed studies open new perspectives for probiotic modulation to specifically antagonizing STEC. Currently, data obtained in vivo and in vitro cannot be extrapolated to humans and only clinical trials in humans will allow the determination of effective probiotic strains and doses.

In conclusion, it is important to investigate the promising role that probiotics could have in modifying the intestinal microbiota of patients with STEC strain infection and to confirm the real efficacy of probiotics in avoiding the onset of or, at least, reducing the severity of HUS.

## Abbreviations

AKI	Acute Kidney Injury
CKD	Chronic Kidney Disease
ESRD	End-Stage Renal Disease
EHEC	Enterohemorrhagic *Escherichia coli*
HUS	Hemolytic Uremic Syndrome
STEC	Shiga-toxin-producing *Escherichia coli*
STX	Shiga toxin
